# Running on a treadmill: dynamic inhibition of APC/C by the spindle checkpoint

**DOI:** 10.1186/1747-1028-2-23

**Published:** 2007-07-24

**Authors:** Laura A Díaz-Martínez, Hongtao Yu

**Affiliations:** 1 Department of Pharmacology, University of Texas Southwestern Medical Center, 6001 Forest Park Road, Dallas, TX 75390-9041, USA

## Abstract

During mitosis, the genome duplicated during S-phase is synchronously and accurately segregated to the two daughter cells. The spindle checkpoint prevents premature sister-chromatid separation and mitotic exit. The anaphase-promoting complex/cyclosome (APC/C) is a key target of the spindle checkpoint. Upon checkpoint activation, the mitotic checkpoint complex (MCC) containing Mad2, Bub3, Mad3/BubR1 and Cdc20 inhibits APC/C. Two independent studies in budding yeast have now shed light on the mechanism by which MCC inhibits APC/C. These studies indicate that Mad3 binds to the mitotic activator of APC/C Cdc20 using peptide motifs commonly found in APC/C substrates and thus competes with APC/C substrates for APC/C^Cdc20 ^binding. In addition, Mad3 binding to APC/C^Cdc20 ^induces Cdc20 ubiquitination by APC/C, leading to the dissociation of MCC. Meanwhile, two other studies have shown that a deubiquitinating enzyme is required for the spindle checkpoint whereas APC/C-dependent ubiquitination is needed for checkpoint inactivation. Collectively, these studies suggest a dynamic model for APC/C^Cdc20 ^regulation by MCC in which APC/C- and Mad3-dependent ubiquitination of Cdc20 constitutes a self-regulated switch that rapidly inactivates the spindle checkpoint upon correct chromosome attachment.

## Background

Accurate chromosome segregation is the key event of mitosis. Errors in this process result in aneuploidy and genome instability, which contributes to cancer progression [1-4]. Mitotic chromosomes consist of pairs of sister chromatids that separate at the onset of anaphase. Sister-chromatid cohesion keeps sister chromatids together from the very moment of chromosome duplication until their separation. At metaphase, sister kinetochores are attached to microtubules emanating from opposite poles, a process referred to as amphitelic attachment or bi-orientation. A multisubunit ubiquitin ligase called the anaphase-promoting complex or cyclosome (APC/C) in conjunction with its mitotic activator Cdc20 then mediates the degradation of cyclin B and securin, allowing the activation of separase, cleavage of cohesin, and equal partition of sister chromatids into the two daughter cells [5,6]. Because microtubule attachment to kinetochores occurs stochastically, improper kinetochore-microtubule attachments, such as syntelic (sister kinetochores attach to microtubules from the same pole), monotelic (only one sister kinetochore attached), and merotelic attachments (a kinetochore attaches to microtubules from both poles), can form during mitosis [7,8]. These improper attachments ought to be corrected prior to sister-chromatid separation. Cells use a control mechanism termed the spindle checkpoint to ensure that all chromosomes are properly attached before initiating chromosome segregation [9,10].

The spindle checkpoint monitors kinetochore-microtubule attachment and possibly inter-kinetochore tension generated by amphitelic attachments [11,12]. The unattached kinetochores are thought to produce diffusible checkpoint signals that inhibit APC/C^Cdc20 ^and block sister-chromatid separation [13,14]. An important checkpoint inhibitor of APC/C is the mitotic checkpoint complex that contains Mad2, Cdc20, Bub3 and BubR1 (Mad3 in budding yeast) [15], although it is presently unclear whether MCC constitutes the diffusible checkpoint signal and whether MCC only forms at the kinetochores [16-19]. Here, we review recent advances in our understanding of APC/C regulation by the mitotic checkpoint complex.

## APC/C Regulation

APC/C is the only known molecular target of the spindle checkpoint, although there is evidence to suggest that other checkpoint targets might exist [20,21]. APC/C-mediated ubiquitination leads to the degradation of cyclin B and securin [5,21,22], allowing efficient sister chromatid separation and mitotic exit. The spindle checkpoint inhibits APC/C and prevents sister-chromatid separation and mitotic exit until all sister chormatids achieve bi-orientation [3,21-25].

In addition to its involvement in anaphase onset and mitotic exit, APC/C regulates other cell cycle events, such as the G1/S transition [26,27] and initiation of DNA replication [28,29]. A growing number of APC/C regulators are required for the precise regulation of APC/C activity during different phases of the cell cycle [5,30-32]. These APC/C regulators can be divided into three categories: (i) APC/C activators, such as Cdc20 and Cdh1, which contribute to substrate recognition and specificity of APC/C [33-39]. (ii) Enzymatic regulators that post-translationally modify core APC/C subunits or its activators [31,40-48]. (iii) APC/C inhibitors, such as MCC [15,49,50] and Emi1 [51,52] that regulate APC/C through direct binding to APC/C or Cdc20 or both.

## MCC, a key checkpoint inhibitor of APC/C^Cdc20^

The first identified checkpoint inhibitor of APC/C was Mad2 [53], which inhibits APC/C through direct binding to Cdc20 [54,55]. The Mad2-Cdc20 interaction is increased during mitosis, when the spindle checkpoint is active [54,56,57]. However, checkpoint inhibition of APC/C^Cdc20 ^turned out to be complex, involving more than the simple Mad2-Cdc20 interaction. In addition to Mad2, Mad3/BubR1-Bub3 binds to Cdc20 directly and inhibits APC/C. Furthermore, Bub1-Bub3 directly phosphorylates Cdc20 and inhibits APC/C^Cdc20^. Although Mad2 and Mad3/BubR1 can inhibit APC/C^Cdc20 ^independently, Mad3/BubR1 potentiates the ability of Mad2 to inhibit Cdc20 [50] and MCC containing BubR1/Mad3, Bub3, Mad2 and Cdc20 inhibits APC/C^Cdc20 ^much more effectively than of Mad2 alone [15]. Furthermore, it has been recently shown that a complex containing Cdc20, BubR1/Mad3 and Mad2 accounts for most of the APC/C inhibitory activity in nocodazole-arrested HeLa cells [58]. Together, these results indicate that MCC is a major checkpoint inhibitor of APC/C. Nonetheless, the existence of MCC sub-complexes indicates that APC/C^Cdc20 ^inhibition by Mad2 and BubR1/Mad3 involves multiple, complex interactions.

## How does MCC inhibit APC/C^Cdc20^?

Because Cdc20 activates the ubiquitin ligase activity of APC/C at least partially through substrate recruitment [36,37,59], it was proposed that MCC interfered with APC/C^Cdc20 ^function by either blocking the access of substrates to Cdc20 or preventing the release of ubiquitinated substrates. Recent studies in budding yeast provide key insights into the mechanism by which MCC inhibits APC/C and establish that the MCC subunit Mad3 (BubR1 in humans) blocks substrate access to APC/C^Cdc20 ^[60,61].

**Figure 1:**
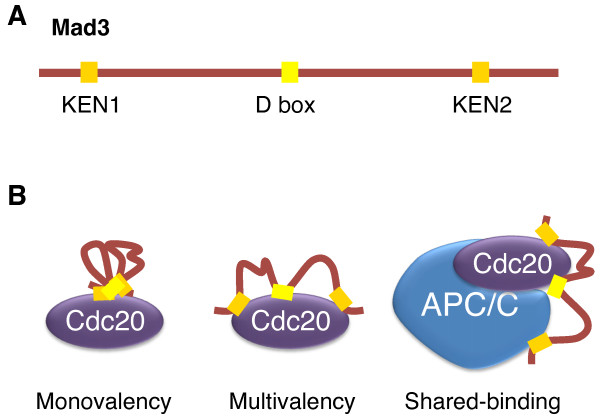
**Mad3 and its interaction with Cdc20**. A. Schematic drawing of *Saccharomyces cerevisiae *Mad3 (homolog of human BubR1). Mad3 contains several APC/C degradation motifs: a destruction box (D box) and two KEN boxes. B. Three possible mechanisms of Mad3 binding to Cdc20. The monovalency model proposes the cooperative binding of multiple motifs into a single docking site in Cdc20. The multivalency model proposes that the multiple degradation motifs of Mad3 bind to different docking sites in Cdc20. In the shared-binding model, the degradation motifs of Mad3 bind to different sites in both Cdc20 and APC/C.

Many APC/C substrates contain short peptide motifs that mediate binding to and ubiquitination by APC/C, including the destruction box (D box) and the KEN box. Two groups have independently discovered that the budding yeast Mad3 protein contains one D box and two KEN boxes (Figure 1A) and that these degradation motifs of Mad3 are required for its spindle checkpoint function [60,61]. Furthermore, mutation of the N-terminal KEN box of Mad3 dramatically reduced the association of Mad3 with Cdc20 and Mad2 *in vivo *[61], indicating that the Mad3 KEN boxes are required for MCC formation. Although Mad3 binds directly to Cdc20 [60], this binding requires Mad2 *in vivo *[60,61]. Addition of Mad2 increases the Mad3-Cdc20 interaction about 4-fold *in vitro *[60]. How Mad2 stimulates Mad3 binding to Cdc20 remains unclear. Mad2 binding could conceivably alter the conformation of Cdc20, allowing more efficient Mad3 binding.

Because mutation of the N-terminal KEN box of Mad3 only reduced, but did not abolish, the Mad3-Cdc20 interaction, Burton and Solomon reasoned that other motifs of Mad3 ought to be involved in Cdc20 binding. Using an *in vitro *peptide competition assay, they determined that the KEN- and D-boxes of Mad3 bind to Cdc20 cooperatively. Furthermore, using the same assay, they showed that Mad3 competes with known Cdc20 substrates for Cdc20 binding, and this ability of Mad3 depends on its D- and KEN-boxes [60]. Taken together, these results indicate that Mad3 inhibits APC/C by blocking substrate binding to APC/C^Cdc20^.

Several non-exclusive models can explain the cooperative binding of KEN and D boxes of Mad3 to Cdc20 (Figure 1B). In the monovalency model, multiple APC/C degradation motifs of Mad3 simultaneously bind to a single substrate-binding site of Cdc20, thus blocking substrate-binding by Cdc20. In the multivalency model, Cdc20 contains multiple substrate-binding sites. Cooperative binding of multiple KEN and D boxes of Mad3 to these sites blocks substrate binding by Cdc20. Finally, in the shared-binding model, Mad3 inhibits APC/C^Cdc20 ^by binding to both Cdc20 and APC/C. This model takes into account the finding that APC/C can directly bind to destruction motifs [62,63]. Simultaneous binding of Mad3 to APC/C and Cdc20 would explain why mutations in the C-terminal KEN box of Mad3 have little effect on Mad3-Cdc20 binding and yet this KEN box is still required for the spindle checkpoint function of Mad3. High-resolution structural studies are required to understand the exact mode of interactions between Mad3 and Cdc20.

## Cdc20 ubiquitination and MCC disassembly

In addition to inhibiting APC/C^Cdc20 ^by blocking substrate recruitment, Mad3 also destabilizes Cdc20 in a spindle checkpoint-dependent manner [64]. King *et al*. demonstrated that destabilization of Cdc20 requires the destruction motifs of Mad3 [61]. It has been recently shown that Cdc20 is ubiquitinated by APC/C when the spindle checkpoint is active [65,66]. Interestingly, Mad3 is unstable in G1 whereas it is stable in nocodazole-treated cells [61]. These results suggest the intriguing possibility that Mad3 binding to Cdc20 directs APC/C activity towards Cdc20, perhaps by mimicking substrate binding. Cdc20 ubiquitination leads to its proteasome-dependent degradation, ensuring that Cdc20 levels are kept below a certain threshold to prevent unscheduled APC/C activation [67].

Two recent studies have shown that degradation of Cdc20 is not the only outcome of Cdc20 ubiquitination [65,66]. Reddy *et al*. have shown that Cdc20 ubiquitination decreases its binding to Mad2 and to APC/C [66]. Cdc20 dissociation from Mad2 and APC/C does not require the proteasome activity and Cdc20 degradation, as proteasome inhibitors did not block this dissociation. Furthermore, addition of the ubiquitin-conjugating enzyme UbcH10, but not its catalytically inactive mutant UbcH10^C114S^, increases both Cdc20 ubiquitination and the dissociation of Cdc20 from Mad2 and APC/C. Conversely, depletion of UbcH10 from HeLa cells decreases APC/C-mediated ubiquitination of Cdc20, stabilizes the Mad2-Cdc20 interaction, and delays anaphase initiation. This study thus establishes that Cdc20 ubiquitination by APC/C is required for checkpoint inactivation and contributes to the dissociation of Cdc20 from Mad2 and possibly the disassembly of MCC[66]. In an accompanying study, Steigmeier *et al*. have identified the ubiquitin-specific protease USP44 as a spindle checkpoint component through an RNA interference (RNAi) screen [65,66]. Human cells depleted for USP44 by RNAi do not undergo mitotic arrest in the presence of spindle poisons. The checkpoint bypass of USP44 RNAi cells depends on APC/C, although Mad2 and BubR1 (Mad3) localize normally to kinetochores. Thus, USP44 acts downstream of Mad2 in APC/C inhibition. Furthermore, USP44 antagonizes APC/C-mediated ubiquitination of Cdc20 *in vivo*. Recombinant USP44 directly deubiquitinates Cdc20 *in vitro *[65]. This study indicates that USP44 reduces Cdc20 autoubiquitination and protects the Mad2-Cdc20-containing checkpoint complexes from disassembly, although it remains to be determined whether USP44 also directly deubiquitinates mitotic APC/C substrates and prevents their degradation.

## The MCC paradox: inhibition through activation?

**Figure 2:**
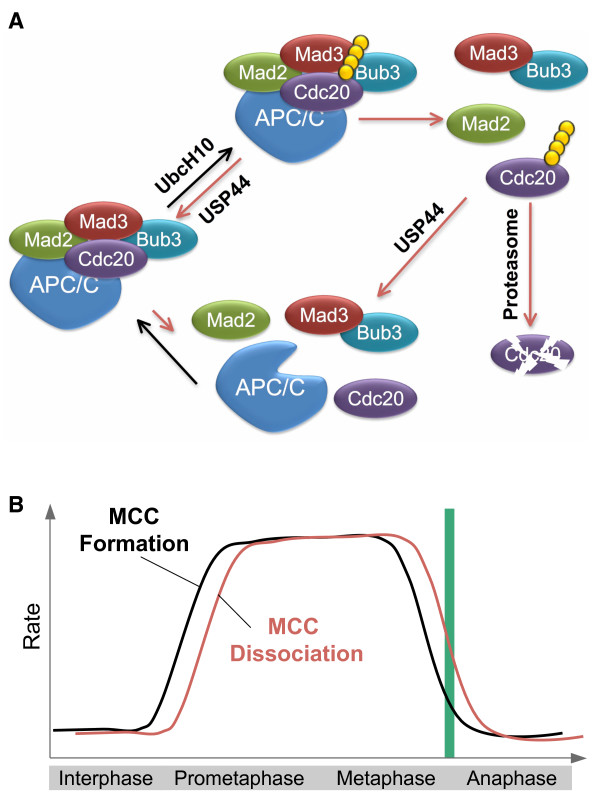
**A dynamic model for MCC-mediated inhibition of APC/C**. A. Mad3 uses its APC/C degradation motifs to bind to Cdc20 and blocks substrate binding of APC/C^Cdc20^. Meanwhile, Mad3 binding to Cdc20 induces APC/C-dependent ubiquitination of Cdc20, which is antagonized by USP44. Ubiquitination of Cdc20 promotes the disassembly of MCC. Ubiquitinated Cdc20 is either degraded by the proteasome to reduce the cellular levels of Cdc20 or deubiquitinated by USP44. The deubiquitinated Cdc20 can be re-incorporated into MCC and associate with APC/C. Thus, upon checkpoint activation, a dynamic equilibrium of MCC formation and disassembly is achieved by the continuous cycles of ubiquitination and deubiquitination of Cdc20. This process directs the activity of APC/C towards Cdc20 and reduces its activity towards cyclin B and securin. B. Schematic drawing of the rates of MCC formation and disassembly during mitosis. Upon checkpoint activation, Mad3 binds to Cdc20 and inhibits APC/C, but Mad3 binding also induces Cdc20 ubiquitination and the disassembly of MCC. Thus, the rates of MCC formation and disassembly may both be enhanced during active spindle checkpoint signaling. An equilibrium is reached to keep the steady-state levels of MCC constant, analogous to a runner on a treadmill. This model is also consistent with the finding that, at any given time, only small pools of the Mad2, Cdc20, Bub3 and Mad3 molecules in a cell associate with APC/C. Once all sister chromatids achieve bi-orientation, the rate of MCC formation falls below that of MCC disassembly. The existing MCC complexes are rapidly disassembled, allowing the activation of APC/C^Cdc20 ^and checkpoint inactivation.

These recent studies establish that, similar to APC/C substrates, Mad3 uses its APC/C degradation motifs to bind to Cdc20, thus blocking substrate binding to APC/C^Cdc20^. Paradoxically, Mad3 binding to Cdc20 also activates the autoubiquitination of Cdc20, which has at least two functions: dissociation of Cdc20 from Mad2 and Cdc20 degradation by the proteasome. Thus, Mad3 binding to Cdc20 would trigger the dissociation of Cdc20 from Mad2. We propose a "dynamic MCC" model to reconcile these findings (Figure 2A). In this model, APC/C inhibition by the spindle checkpoint is achieved through a regulated, dynamic equilibrium of Cdc20 ubiquitination and deubiquitination. Checkpoint activation inhibits APC/C^Cdc20 ^by enhancing the formation of MCC and possibly its association with APC/C. Mad3 blocks access of substrates to APC/C^Cdc20 ^and simultaneously induces Cdc20 ubiquitination. In human cells, Cdc20 ubiquitination is stimulated by UbcH10 and possibly p31^comet ^and is reversed by USP44. Cdc20 ubiquitination leads to Cdc20 dissociation from both APC/C and MCC components. Ubiquitinated Cdc20 is either degraded by the proteasome or deubiquitinated by USP44. If the spindle checkpoint stays on, MCC can re-assemble and bind to and inhibit the APC/C activity toward other substrates. Upon the proper attachment of the last chromosome to the mitotic spindle, the existing MCC can be rapidly disassembled through MCC- and APC/C-dependent ubiquitination of Cdc20, leading to checkpoint inactivation.

The steady state levels of MCC are determined by the rates of MCC formation and disassembly. Three possible scenarios can explain the higher concentrations of MCC in mitosis. First, the rate of MCC formation increases during mitosis whereas the rate of MCC disassembly remains constant during the cell cycle. Second, an increase in the rate of MCC formation during mitosis is accompanied by a concomitant drop in the rate of MCC disassembly. The "dynamic MCC" model predicts a third scenario of MCC regulation, in which both rates of MCC formation and disassembly increase during mitosis (Figure 2B). By being dynamic, the levels of MCC are more responsive to the status of checkpoint signaling. Once the sister chromatids achieve proper attachment, MCC can be quickly inactivated to allow for APC/C activation and sister-chromatid separation. In contrast, the first two scenarios predict a slower and undesirable process of MCC inactivation and checkpoint silencing.

## Perspective

Recent discoveries establish a mechanism for Mad3-dependent inhibition of APC/C^Cdc20 ^and reveal the function and regulation of a dynamic Cdc20 ubiquitination/deubiquitination cycle during mitosis. These studies support a "dynamic MCC" model for maintaining a robust spindle checkpoint and for the mechanistic coupling between APC/C inhibition and checkpoint inactivation. Many questions remain unresolved: does BubR1 (the vertebrate ortholog of Mad3) bind to Cdc20 and inhibit APC/C^Cdc20 ^using a mechanism similar to Mad3? Is USP44 conserved in other organisms? Is the activity of USP44 regulated by the spindle checkpoint? How are degradation of Cdc20 and its deubiquitination by USP44 coordinated? Future research aimed at addressing these questions will further advance the molecular understanding of the spindle checkpoint.

## Competing interests

The author(s) declare that they have no competing interests.

## Authors' contributions

LADM and HY wrote the paper. All authors read and approved the final manuscript.
